# Creating a supportive legal environment for universal health coverage

**DOI:** 10.2471/BLT.16.173591

**Published:** 2016-07-01

**Authors:** David Clarke, Dheepa Rajan, Gerard Schmets

**Affiliations:** a Department of Health Systems Governance, Policy and Aid Effectiveness, World Health Organization, avenue Appia 20, 1211 Geneva 27, Switzerland.

In this edition of the *Bulletin*, Marks-Sultan et al.^1^ propose that the World Health Organization (WHO) should provide capacity-building for drafting health laws in Member States. They highlight that WHO has the authority and credibility to work with countries to make their national laws easier to access, understand, monitor and evaluate. WHO’s new technical support work related to universal health coverage (UHC) laws is a good example of its support for Member States in this important area.

Strengthening countries’ legal and regulatory frameworks and engaging in universal health coverage-compliant law reforms has been missing from the universal health coverage agenda. WHO calls on Member States to align their health system policies with universal health coverage goals such as equity, efficiency, health service quality and financial risk protection. Strengthening health systems using health laws and legal frameworks is a pivotal means for attaining these goals^2^ and achieving sustainable results in health security and resilience.

Laws are needed to ensure the equity, quality and safety of health services and financial protection for health system users. A strong legal framework sets the rules for how the health system functions, establishes a legal mandate for access to health services and provides the means by which a national government can implement universal health coverage at a population level. Several governments have already successfully used their health laws in service of their universal health coverage goals. For example, the governments of the Bolivarian Republic of Venezuela, Brazil, Chile, Colombia, Cuba, Mexico, Peru and Uruguay have all legislated a right to health, which entitles their citizens to expanded access to health services.

To date, countries’ legal and regulatory frameworks have not been systematically assessed for their compatibility with the goals of universal health coverage. Work on law reform so far has only focused on individual laws rather than on creating a supportive legal environment for universal health coverage. In many countries, information is lacking on the extent to which existing national laws support or block the goals of universal health coverage.^3^ A different approach is required. WHO thus recommends that countries analyse their existing legal and regulatory frameworks at international, national and local level and assess their compatibility with universal health coverage, with the ultimate aim of ensuring a legal environment that supports the functioning of the entire health system. 

In practice, the work on universal health coverage law reform is highly technical and political. It involves legal analysis, multi-stakeholder dialogue and policy discussions.^4^ A government’s capacity to make and enforce laws and its broader political and economic situation, heavily influence whether law reform can happen. Technical law makers alone cannot address these issues. For example, in Turkey, a universal health coverage reform process included policy dialogue and reflection on the necessary supporting legal reform needed and this consultation led to an accelerated universal health coverage reform implementation.^5^ Turkey’s experience demonstrates the centrality of law-related policy discussions and multi-stakeholder dialogue on law reform and their importance for the ultimate success of UHC reform.

WHO’s technical support to Member States on universal health coverage laws should similarly take a systems approach. In response to requests from its Member States, WHO is working to help governments create supportive legal environments for universal health coverage through legal environment assessments, specialist technical support, multi-stakeholder policy dialogue and advice and guidance on law reform.
